# Uptake, knowledge, attitudes, and practices toward seasonal influenza vaccination among community healthcare workers during the COVID-19 pandemic in Chongqing Municipality, China: A cross-sectional study

**DOI:** 10.1371/journal.pone.0327012

**Published:** 2025-07-03

**Authors:** Xianxian Yang, Jiajing Zheng, Xiaoyan Lv, Qing Wang, Dong Wang, Jiaxi Xie, Xianbin Ding, Ting Chen

**Affiliations:** 1 Chongqing Municipal Center for Disease Control and Prevention, Chongqing, China; 2 School of Public Health, Chongqing Medical University, Chongqing, China; 3 Information Technology Center, West China Hospital, Sichuan University, Chengdu, Sichuan Province, China; Universitas Syiah Kuala, INDONESIA

## Abstract

**Background:**

Seasonal influenza is significantly associated with high morbidity and mortality. Vaccination of healthcare workers (HCWs), who represent a high-risk group, is a crucial preventive measure to reduce the spread of outbreaks and the severity of disease. Therefore, the aim of this study was to evaluate the levels of influenza vaccine acceptance and uptake, as well as the determinants influencing these factors among HCWs in Chongqing, China.

**Methods:**

We conducted a cross-sectional survey among community HCWs aged ≥ 22 years from July 15, 2021 to September 30, 2021. An anonymous self-administered questionnaire was used to assess the knowledge and key factors of HCWs regarding vaccination acceptance and recommendation to patients. Parametric and nonparametric statistical analyses were performed with the use of the Statistical Program for the Social Sciences (SPSS) version 25.0.

**Result:**

The coverage rate for the seasonal influenza vaccination in Chongqing HCWs was 46.2% in the 2020–2021 seasons. HCWs who were females (OR: 2.10 95% CI: 1.54–2.85), had influenza experience (OR:1.98 95% 1.27–3.11), without influenza vaccination hesitancy (IVH) (OR:2.10 95% 1.58–2.79), and/or in a vaccination-promoted community (OR:6.50 95% 3.99–10.60) would be more likely to be vaccinated. HCWs who were vaccinated in 2020–2021 (OR: 8.48 95% 6.03–11.93), without IVH (OR: 4.15 95% 2.91–5.90), and/or in a vaccination-promoted community (OR: 2.08 95%1.37–3.16) would be more willing to be vaccinated. HCWs who were with knowledge scores ≥ 4 (OR: 2.58 95% 1.30–5.13), without IVH (OR: 2.33 95% 1.05–5.13), were in a vaccination-promoted community (OR: 2.65 95% 1.51–4.65), and/or with vaccination willingness (OR: 4.32 95% 2.16–8.64) would be more likely to recommend vaccination to patients.

**Conclusion:**

The influenza vaccination rate among Chongqing HCWs increased in the 2020–2021 season but remained relatively low. It is critical to improve knowledge of influenza and its vaccine among HCWs, implement activities to moderate their IVH and expand the free vaccination policy, and interventional measures aiming to promote influenza vaccination.

## Introduction

Seasonal influenza is an acute respiratory disease caused by influenza type A and B viruses [1], which was associated with significant morbidity and mortality even before the coronavirus disease 2019 (COVID-19) pandemic [2]. According to the World Health Organization (WHO), the annual global attack rate of seasonal influenza is estimated at 5−10% in adults and 20−30% in children, resulting in three to five million cases of severe illness and between 290,000 and 650,000 deaths [3]. HCWs play a crucial role in influenza transmission dynamics, being both at elevated risk of infection and potential vectors for transmission to vulnerable patient populations [4]. Influenza vaccination is a critical strategy for mitigating these risks, demonstrating efficacy in reducing outbreak incidence and disease severity. It is universally acknowledged as the primary preventive measure against influenza and its associated health and economic burdens [5]. In China, influenza vaccination coverage has historically remained suboptimal, with a notably low rate of 0.62% during the 2018−2019 influenza season [6]. The COVID-19 pandemic appears to have influenced vaccination behaviors, with coverage rates increasing to approximately 3% in subsequent seasons [6]. This upward trend suggests that the pandemic may have enhanced public awareness and acceptance of vaccination as a preventive health measure. Nevertheless, China’s vaccination coverage remains significantly lower than that of many other countries or regions [7,8].

Existing research indicates that the influenza vaccination rate among HCWs varies significantly across different countries and regions. In the United States, influenza vaccine coverage among HCWs has surpassed 75% [9], while a survey reporting official vaccination coverage rates in 10 European countries revealed that uptake among HCWs has consistently remained below 35% [10]. Alarmingly, the influenza vaccination rate among Chinese medical staff was only 11.6% during the 2018–2019 period [11], with regional estimates of vaccination coverage among HCWs in China ranging from 5% to 18% [11–13], largely due to differences in subsidy policies [14]. Furthermore, vaccine hesitancy—defined by the World Health Organization (WHO) as the delay or refusal to vaccinate despite the availability of vaccination services [15], is prevalent among HCWs and has been recognized as a significant global public health threat [16,17]. As trusted sources of health information [18–20], the attitudes and vaccination behaviors of HCWs directly impact public perceptions and intentions regarding vaccination. When HCWs exhibit vaccine hesitancy, it can undermine public trust in the safety and efficacy of vaccines, potentially resulting in decreased vaccination rates among the general population [21]. Moreover, most existing studies on influenza vaccination rates and the factors influencing them have focused primarily on developed countries, leaving a substantial research gap regarding low- and middle-income countries [22]. This gap is particularly pronounced in the context of the COVID-19 pandemic, during which there has been limited research on the knowledge, attitudes, and practices (KAP) of HCWs concerning the influenza vaccine. The lack of comprehensive studies in this area restricts our understanding of vaccine hesitancy among HCWs and hinders the development and implementation of interventions aimed at increasing vaccination rates.

Although the significance of influenza vaccination for HCWs and patients is widely acknowledged, data on influenza vaccination rates and the of HCWs in specific regions of China, such as Chongqing municipality, remain limited. The COVID-19 pandemic has further underscored the importance of influenza vaccination, as the co-occurrence of influenza and COVID-19 can place additional strain on public health systems. Therefore, understanding the vaccination behaviors of HCWs and the factors influencing these behaviors is essential for developing targeted intervention strategies and improving the vaccination rates. In the context of COVID-19, studying the KAP of HCWs regarding the influenza vaccine holds significant practical implications.

The primary objectives of this study were to evaluate the influenza vaccination rate and its influencing factors among HCWs in Chongqing during the COVID-19 pandemic and to analyze their KAP regarding the influenza vaccine. We hypothesized that HCWs’ perceptions of influenza and its vaccination are significantly associated with their vaccination behaviors, and that the COVID-19 pandemic may have increased the acceptance of influenza vaccination among HCWs. By addressing gaps in existing research, this study aims to provide scientific evidence for the development of targeted interventions to enhance the influenza vaccination rates among HCWs and the general public.

## Materials and methods

### Study design and population

Chongqing is one of the four municipalities directly under the Central government of China, which is located in southwestern China. The Chongqing municipality consists of 26 districts and 12 counties (the districts and counties are of the same administrative level in Chongqing), which has a population of 34.1 million residents and 14,909 healthcare workers registered in 2021 [23].

This cross-sectional survey was based on self-reported data collected through an online questionnaire survey powered by Wenjuanxing (wjx. cn) in Chinese version, from 15 July 2021 to 30 September 2021. Participants were sampled by multi-stage stratified random sampling. Firstly, we randomly selected 6 of 38 districts and counties according to social economic, population composition, geographical location, and accessibility. Secondly, we randomly selected 10 street (township) community health service centers (CHSCs) in each district/county. Then, we randomly selected 20 participants (HCWs providing direct patient care) from each CHSC or township health center. Among the 1,200 HCWs initially recruited for the study, 1,142 completed the survey questionnaires, achieving a primary response rate of 95.2%. Following data quality assessment, 112 responses were excluded due to incompleteness or inconsistency, resulting in 1,030 valid responses for final analysis. This corresponds to an overall inclusion rate of 85.8% in the final analytical sample.

The inclusion criteria were all community HCWs in 60 CHSCs and aged ≥ 22 years. We defined community HCWs as those who worked at community health centers providing primary health services, including general practitioners, public health physicians, nurses working on the front line, and public health workers (both doctors and nurses) responsible for vaccination. Participants were excluded from the final analysis if they met any of the following criteria: (1) incomplete responses, (2) duplicate entries, (3) non-eligibility based on predefined criteria. Community HCWs enrolled in this study with no incentives for participation and completed self-administered electronic questionnaires. All participants had written informed consent before the study.

The minimum required sample size of 1067 respondents were calculated using the formula: $\;N=(Z^{2}\times P\times (1-P))/d^{2}$, which was used for the calculation. Among them, Z is the value from the standard normal distribution corresponding to the desired confidence level (Z = 1.96 for 95% CI), *P* represents the estimated proportion (*P* = 50%), and d stands for the desired precision of estimate (margin of error) (d = 3%).

### Data collection

An anonymous self-administered questionnaire was used to assess the knowledge and key factors that underlie the HCWs’ practices related to acceptance of vaccination and recommendations to patients. The creation of the questionnaire was based on previously relevant literature [24–27]. The questionnaire was composed of the following four sections from Part I–IIII: Part I showed the personal socio-demographic status (age, sex, residence, education level, and perceived health status). Part II showed information about KAP regarding influenza and influenza vaccines. Part III covered detailed information on influenza vaccine hesitancy. Part Ⅳ collected information on the measures proposed by medical staff on how to improve influenza vaccination coverage among community HCWs. Open-ended questions focused on reasons for refusing vaccination, willingness to vaccinate, perceptions of barriers to influenza vaccination, the willingness and trust in institutions to recommend vaccines to patients, and the measures proposed by medical staff on how to improve influenza vaccination coverage among community HCWs. More details are provided in the supplementary file 1 questionnaire. In addition, we obtained the vaccination status of the participants in the 2019–2020 season from the Chongqing Municipal Immunization and Prevention System.

To ensure the quality of the collected data, this study was first conducted as a small-scale pilot questionnaire-based survey, encompassing 30 samples from eight districts and counties. After this pretest, the questionnaire was modified by experts in related fields.

The data from the final questionnaires were analyzed statistically. Based on Cronbach’s alpha and factor analysis, the questionnaire has high validity and reliability: Cronbach’s alpha reliability coefficient was 0.849, and the KMO validity coefficient was 0.894 in Part III. These results confirmed the validity and reliability of the questionnaire.

### Survey instruments

Knowledge about influenza disease and vaccines, including 6 questions: (1) Influenza is different from the common cold; (2) The whole population is susceptible to influenza; (3) Influenza vaccine can only prevent influenza annually; (4) Influenza can be spread through respiratory droplets, or direct or indirect contact with mucous membranes such as the mouth, nose and eyes attitudes toward influenza vaccines, and the practice related to influenza and influenza vaccines; (5) What do you think is the coverage rate of influenza vaccine for the whole population in China; (6) Influenza vaccination can protect themselves as well as their families and patients for HCWs.

Attitudes toward influenza vaccine, including 3 questions: (1) Community HCWs should receive influenza vaccine. (2) Flu vaccines should be covered by public health insurance. (3) The government should introduce a policy to provide free flu vaccinations for healthcare workers and other eligible groups. Practice related to influenza and influenza vaccines, including 5 questions: (1) Have you had an influenza-like illness (ILI) in the past 3 years? (2) Have you had influenza in the past 3 years? (3) Have you asked for leave due to influenza or ILI in the past 3 years? (4) Did you receive the influenza vaccine in the last flu season? (5) How do you pay for influenza vaccination?

### Study measures

The major outcome measures in this study were: (1) influenza vaccine uptake in the last influenza season (yes vs. no); (2) willingness to get influenza vaccination in the next influenza season (yes vs. no/maybe); and (3) willingness to recommend the vaccine to the patients (yes vs. no/maybe). The covariates were sex, age (≥30 years vs. < 30 years), residence (urban vs. rural), educational level (university and above education vs. and below), professional qualifications (senior title vs. and below), years engaged in medical service (≥10 years vs. < 10 years), and perceived health status (good vs. and below), influenza experience was defined influenza experience as: had influenza in the past 3 years. (yes vs. no). Knowledge about influenza disease and vaccines, including influenza disease severity, transmission, benefits of vaccination and recommendations. Based on these responses, we calculated a knowledge index score for HCWs. The score was calculated based on correct (one point) and incorrect (zero points) responses to 6 statements, the median index score and the interquartile range (IQR) were reported, and we defined the knowledge score (≥ 4 vs. < 4). Influenza vaccination hesitancy (yes vs. no), the hospital has actively promoted vaccination of medical staff (yes vs. no). Influenza vaccination hesitancy (IVH) was defined as knowing that influenza vaccine and vaccination services are available, but not quite sure whether to get vaccinated or remain concerned after vaccination. Options included: (1) completely reject, (2) reject but still considering, (3) have not decided yet or never thought about it, (4) accept but still considering, and (5) completely accept. Respondents who chose options 2, 3, or 4 were considered to have IVH. More details are provided in our previous study [28].

### Statistics analysis

Statistical analyses were performed using the Statistical Program for Social Sciences (SPSS) version 25.0 (IBM Corporation, New York, NY, USA). A descriptive statistical method was used to calculate vaccination coverage, the number of influenza vaccination payment methods, measures for improving influenza vaccination coverage, and other variables. Categorical variables were compared using the Pearson chi-squared test. The independent sample t-test was used to evaluate the differences in the coverage rates for the seasonal influenza vaccination in community HCWs between the 2019–2020 season and the 2020–2021 season. Logistic regression analysis (forward-stepwise-regression) was used to explore relative factors of influenza vaccine uptake, willingness to be vaccinated, and willingness to recommend influenza vaccination in patients, after adjusting for potential confounding variables based on the odds ratios (OR) and 95% confidence interval (CI). Participants who didn’t get vaccinated during the 2020–2021 season, participants who didn’t have the willingness to be vaccinated in the next influenza season, and participants who didn’t have the willingness to recommend vaccination to their patients were considered as the reference group were respectively considered as the reference group. Potential confounding factors, including sociodemographic status (age, gender, education level, professional qualifications, years engaged in medical service, self-reported health conditional), experiences (had influenza in the past 3 years versus not, vaccinated in 2020–2021 versus not vaccinated), the score of knowledge (≥ 4 vs. < 4), IVH and the willingness to be vaccinated in the next influenza season. All statistical tests were two-sided, and a *P* value < 0.05 was considered statistically significant.

### Ethical approval

The study protocol and questionnaire in Chinese version were approved by the Research Ethics Committee of the Chongqing Center for Disease Control and Prevention (KY-2022-014-2). The participants were reassured of the confidentiality of the collected information and signed informed consent and that they could withdraw from the study at any time.

## Results

### Demographics

The coverage rate for the seasonal influenza vaccination in HCWs was 46.2% (95 CI: 43.2%−49.3%) in the 2020−2021 season during the COVID-19 pandemic. Women accounted for 71.6% (737) of the respondents, and 293 questionnaires (28.4%) were completed by men. Most participants were between 30 and 49 years old (790 participants; 76.7%). 54.7% of participants lived in the rural area. Among the participants, 15.05% had a high/secondary school or lower degree. Nursing accounted for the greatest number of respondents (341 participants; 33.1%) followed by clinical medicine (323 participants; 31.4%), integrative medicine (116 participants; 11.3%), and the others (120 participants; 11.6%). 85 (8.2%) participants were in traditional Chinese medicine, and 45 (4.4%) were in preventive medicine/public health. Moreover, most participants had at least 5 years of experience in healthcare; 487 (47.3%) and 377 (36.6%) participants had 5–20 and ≥ 20 years of experience, respectively. There was significantly more uptake of the vaccine among female HCWs compared to males (*p* < 0.001). The significant differences also existed in the characteristics of highest education level, highest degree major, professional qualifications, perceived health status, and influenza experience between the vaccination uptake subgroups (*p* < 0.05). Age(*p* = 0.249), residential status(*p* = 0.201), and years of medical service(*p* = 0.085) did not show any statistically significant effect on vaccine uptake Table 1.

**Table 1:** Frequency table of demographic characteristics of community HCWs categorized by vaccination uptake.

Characteristics	Study population n = 1030		Vaccine taken n = 476 (46.2%)		*P*
	N	%	N	%	
**Gender**					<0.001
Male	293	28.45	97	33.1	
Female	737	71.55	379	51.4	
**Age (year)**					0.249
<30	240	23.30	101	42.1	
30-	317	30.78	145	45.7	
40-	473	45.92	230	48.6	
**Residence**					0.201
Urban	467	45.34	226	48.4	
Rural	563	54.66	250	44.4	
**Highest education level**					0.001
High/secondary school or lower	155	15.05	50	32.3	
Junior college	377	36.60	181	48.0	
Bachelor or higher	498	48.35	245	49.2	
**Highest degree major**					0.001
Clinical	323	31.36	136	42.1	
Traditional Chinese medicine	85	8.25	42	49.4	
Integrative Medicine	116	11.26	35	30.2	
Nursing	341	33.11	179	52.5	
Preventive medicine/public health	45	4.37	23	51.1	
Other	120	11.65	61	50.8	
**Years of medical service**					0.085
<5	166	16.12	63	38.0	
5-10	220	21.36	104	47.3	
10-15	210	20.39	91	43.3	
15-20	57	5.53	29	50.9	
>20	377	36.60	189	50.1	
**Professional title** ^a^					<0.001
Primary or lower	693	67.28	291	42.0	
Middle	265	25.73	142	53.6	
Senior	72	6.99	43	59.7	
**Perceived health status**					0.017
Good	763	74.08	333	43.6	
General	187	18.16	98	52.4	
Fair/Poor	80	7.77	45	56.3	
**Influenza experience**					<0.001
Yes	109	10.58	68	62.4	
No/not be sure	921	89.42	408	44.3	

Note: The chi-square test and *p*-value refer to the comparison of the vaccine taken vs the vaccine not taken group.

^a^Primary: equals to a resident physician; Middle: equals to Chief physician; Senior: equals to Professor.

### Influenza vaccination status

**Fig 1:**
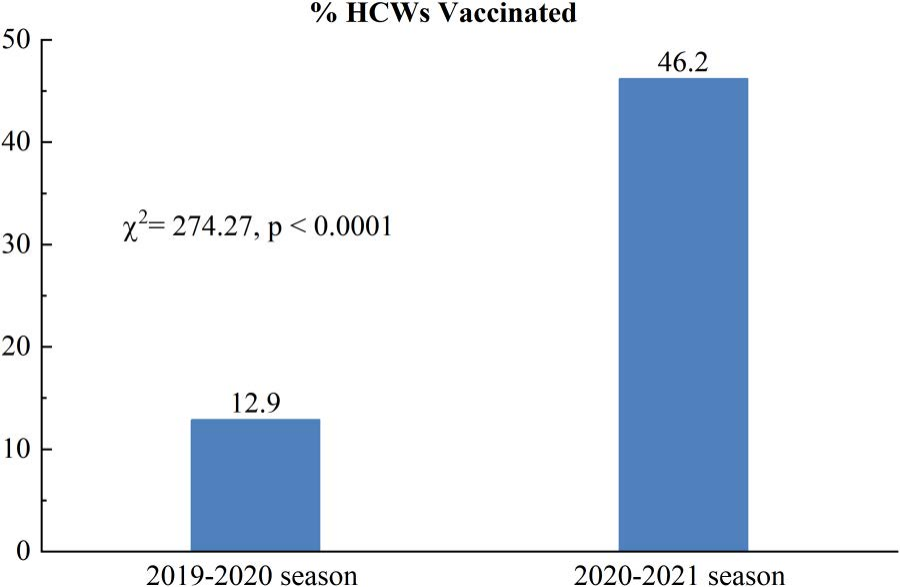
HCWs’ influenza vaccination status.

The coverage rates for the seasonal influenza vaccination in community HCWs were 12.9% (95 CI: 10.9%−15%) in the 2019−2020 season and 46.2% (95 CI: 43.2%−49.3%) in the 2020−2021 season during the COVID-19 pandemic. The coverage of HCWs showed significant differences in the two years before and after the COVID-19 pandemic (^2^ = 274.27, *p* < 0.0001) (Fig 1).

A total of 476 respondents got vaccinated during the 2020–2021 season. Among them, 62.6% (298/476) of respondents chose to receive the 4-valent influenza vaccine, and 178 (37.4%) chose the 3-valent influenza vaccine. The most common payment method was free of charge, accounting for 39.9% (190/476) of respondents, followed by self-payment (37.4%, 178/476) and medical insurance payment (19.7%, 94/476) (S1 Fig).

### Knowledge about influenza vaccine

**Fig 2:**
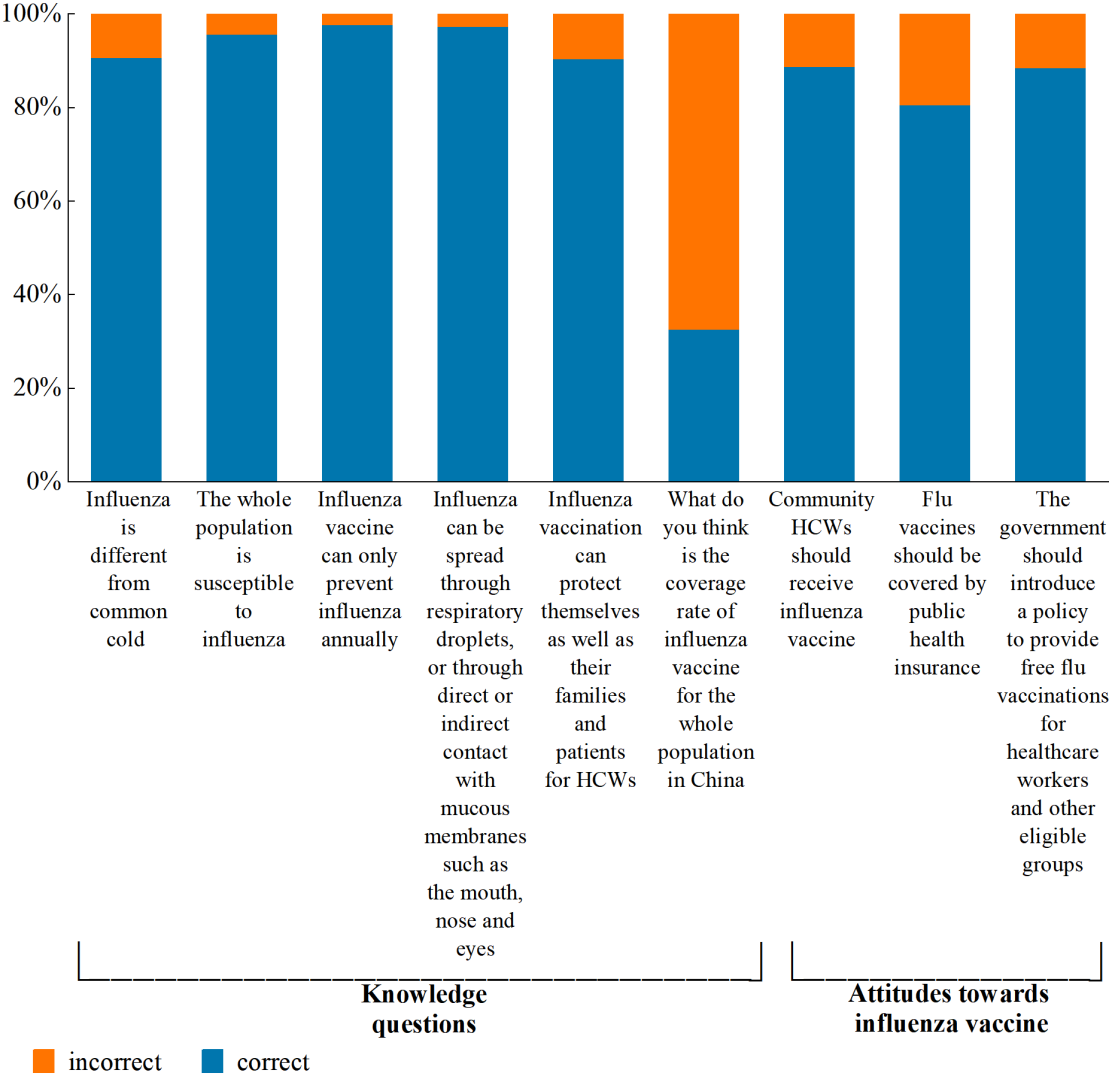
Knowledge and attitude towards influenza vaccine.

The mean knowledge score for participants was 4.2/6.0 (SD: 0.8). with a median score of 4 (IQR: 4–5). Among the respondents, only 33.2% had a knowledge score greater than the mean. Vaccinated HCWs had no significant (knowledge score ≥ 4) than those who were unvaccinated (knowledge score < 4) during the 2020–2021 season (*p* > 0.05). 90.7% of respondents (n = 934) did not believe influenza differs from the common cold, and 4.4% (n = 45) did not believe the whole population is susceptible to influenza. Although influenza is spread through respiratory droplets, 97.7% (n = 1003) of the respondents believed influenza could spread through direct or indirect contact with mucous membranes such as the mouth, nose, and eyes. 2.3% (n = 24) of the respondents claimed that the influenza vaccine for HCWs would not prevent influenza, while 32.7% (n = 337) of the respondents knew coverage rate of influenza vaccine for the whole population in China. 90.5% (n = 932) indicated that the influenza vaccine can protect themselves as well as their families and patients for HCWs (Fig 2).

### Association between socio-demographic factors, knowledge scores, attitudes, and influenza vaccine uptake in the 2020–2021 season

In the bivariate influenza analysis, females (OR: 2.14, 95% CI: 1.61–2.84), participants with a senior title professional qualifications (OR: 1.80, 95% CI: 1.10–2.93), HCWs who had influenza experience (OR: 2.09, 95% CI: 1.39–3.14), HCWs without IVH (OR: 2.08, 95% CI: 1.60–2.70), and HCWs who were in an active vaccination promoted Community Health Services (CHS) (OR: 6.33, 95% CI: 3.95–10.13) were significantly more likely to be vaccinated for influenza in the 2020–2021 season. Participants with a university or postgraduate degree were less willing to receive vaccination (OR: 1.26, 95% CI: 0.99–1.61), compared to participants with technical or undergraduate degrees only, though this finding was not statistically significant. After adjusting for potential confounders in the model, compared with those not vaccinated during the 2020–2021 season, the adjusted ORs (95% CIs) of the variables are as follows: HCWs who were females (adjusted OR: 2.10, 95% CI: 1.54–2.85), HCWs who had influenza experience (adjusted OR: 1.98, 95% CI: 1.27–3.11), HCWs without IVH (adjusted OR: 2.10, 95% CI: 1.58–2.79), and HCWs who were in an active vaccination promoted CHS (adjusted OR: 6.50, 95% CI: 3.99–10.60) were significantly more likely to be vaccinated for influenza in the 2020–2021 season. Yet, HCWs with a perceived health status (adjusted OR: 0.58, 95% CI: 0.35–0.97) had not a positive attitude toward vaccination Table 2.

**Table 2:** The association of socio-demographic factors with knowledge, attitudes, and practice of influenza vaccination.

Characteristics	Influenza vaccine uptake				Willingness to vaccinate#				Recommend to patients			
	Unadjusted OR (95% CI)	*P*	Adjusted OR (95% CI)	*P*	Unadjusted OR (95% CI)	*P*	Adjusted OR (95% CI)	*P*	Unadjusted OR (95% CI)	*P*	Adjusted OR (95% CI)	*P*
Females versus males ^*^	2.14 (1.61-2.84)	<0.001	2.10 (1.54-2.85)	<0.001	1.70 (1.29-2.23)	<0.001	1.31 (0.93-1.84)	0.122	0.82 (0.49-1.37)	0.445		
Age ≥ 30 years versus < 30 years ^*^	1.24 (0.93-1.67)	0.143	1.08 (0.69-1.68)	0.773	1.10 (0.82-1.47)	0.548	0.89 (0.54-1.49)	0.667	0.89 (0.51-1.55)	0.672	0.89 (0.39-2.03)	0.783
University and above education versus and below ^*^	1.26 (0.99-1.61)	0.063	1.18 (0.90-1.56)	0.235	0.91 (0.70-1.17)	0.440	0.70 (0.51-0.96)	0.027	0.87 (0.54-1.41)	0.567	1.17 (0.69-1.97)	0.563
Professional title (senior title versus and below ^*^)	1.80 (1.10-2.93)	0.018	1.72 (0.99-3.00)	0.055	1.18 (0.72-1.96)	0.512	0.85 (0.45-1.60)	0.604	1.01 (0.39-2.59)	0.986	0.85 (0.30-2.39)	0.765
≥10 years of service versus <10 years of service ^*^	1.21 (0.94-1.56	0.142	1.33 (0.90-1.98)	0.155	1.17 (0.90-1.52)	0.235	1.47 (0.93-2.32)	0.098	0.92 (0.56-1.50)	0.724	1.21 (0.57-2.53)	0.632
Perceived Health status (good versus and below ^*^)	0.65 (0.41-1.02	0.062	0.58 (0.35-0.97)	0.036	1.14 (0.72-1.81)	0.579	1.40 (0.78-2.51)	0.257	0.64 (0.30-1.39)	0.257	1.49 (0.65-3.45)	0.349
Influenza experience (yes versus no ^*^)	2.09 (1.39-3.14)	<0.001	1.98 (1.27-3.11)	0.003	1.24 (0.82-1.88)	0.318	0.87 (0.52-1.47)	0.609	0.62 (0.25-1.58)	0.319	1.44 (0.55-3.81)	0.46
Knowledge scores ≥4 versus < 4^*^	0.91 (0.60-1.38)	0.651	0.72 (0.45-1.14)	0.156	1.08 (0.70-1.66)	0.728	1.04 (0.62-1.76)	0.882	2.66 (1.42-4.99)	0.002	2.58 (1.30-5.13)	0.007
IVH (no versus yes ^*^)	2.08 (1.60-2.70)	<0.001	2.10 (1.58-2.79)	<0.001	4.21 (3.10-5.74)	<0.001	4.15 (2.91-5.90)	<0.001	4.43 (2.10-9.34)	<0.001	2.33 (1.05-5.14)	0.037
Hospital has actively promoted vaccination of medical staff (yes versus no ^*^)	6.33 (3.95-10.13)	<0.001	6.50 (3.99-10.60)	<0.001	3.80 (2.64-5.47)	<0.001	2.08 (1.37-3.16)	0.001	4.39 (2.63-7.33)	<0.001	2.65 (1.51-4.65)	0.001
Vaccinated in 2020–2021 versus not vaccinated ^*^					9.80 (7.16-13.43)	<0.001	8.48 (6.03-11.93)	<0.001	4.17 (2.25-7.70)	<0.001	1.48 (0.72-3.04)	0.287
Willingness to be vaccinated in the next season (yes versus no ^*^)									7.48 (4.11-13.62)	<0.001	4.32 (2.16-8.64)	<0.001

# Indicates the next influenza season (2021–2022).

* Indicates the reference group.

### The association between socio-demographic factors, knowledge scores, and attitudes with the willingness to be vaccinated

In the bivariate analysis, females (OR: 1.70, 95% CI: 1.29–2.23), participants vaccinated in the 2020/2021 season (OR: 9.80, 95% CI: 7.16–13.43), HCWs without IVH (OR: 4.21, 95% CI: 3.10–5.74), and HCWs who were in an active vaccination promoted CHS (OR: 3.80, 95% CI: 2.64–5.47) were significantly greater inclination to receive the vaccine. After adjusting for potential confounders in the model, compared with not willingness to be vaccinated in the next influenza season, the adjusted ORs (95% CIs) of the variables are as follows: participants with a postgraduate degree were significantly less willing to receive the vaccine (adjusted OR: 0.70, 95% CI: 0.51–0.96). Meanwhile, participants vaccinated in the 2020–2021 season (adjusted OR: 8.48, 95% CI: 6.03–11.93), participants without IVH (adjusted OR: 4.15, 95% CI: 2.91–5.90), and HCWs who were in an active vaccination promoted CHS (adjusted OR: 2.08, 95% CI: 1.37–3.16) remained a significantly higher willingness to receive the vaccine in the next influenza season Table 2.

### The association between socio-demographic factors, knowledge scores, and attitudes with the willingness to recommend influenza vaccination in patients

**Fig 3:**
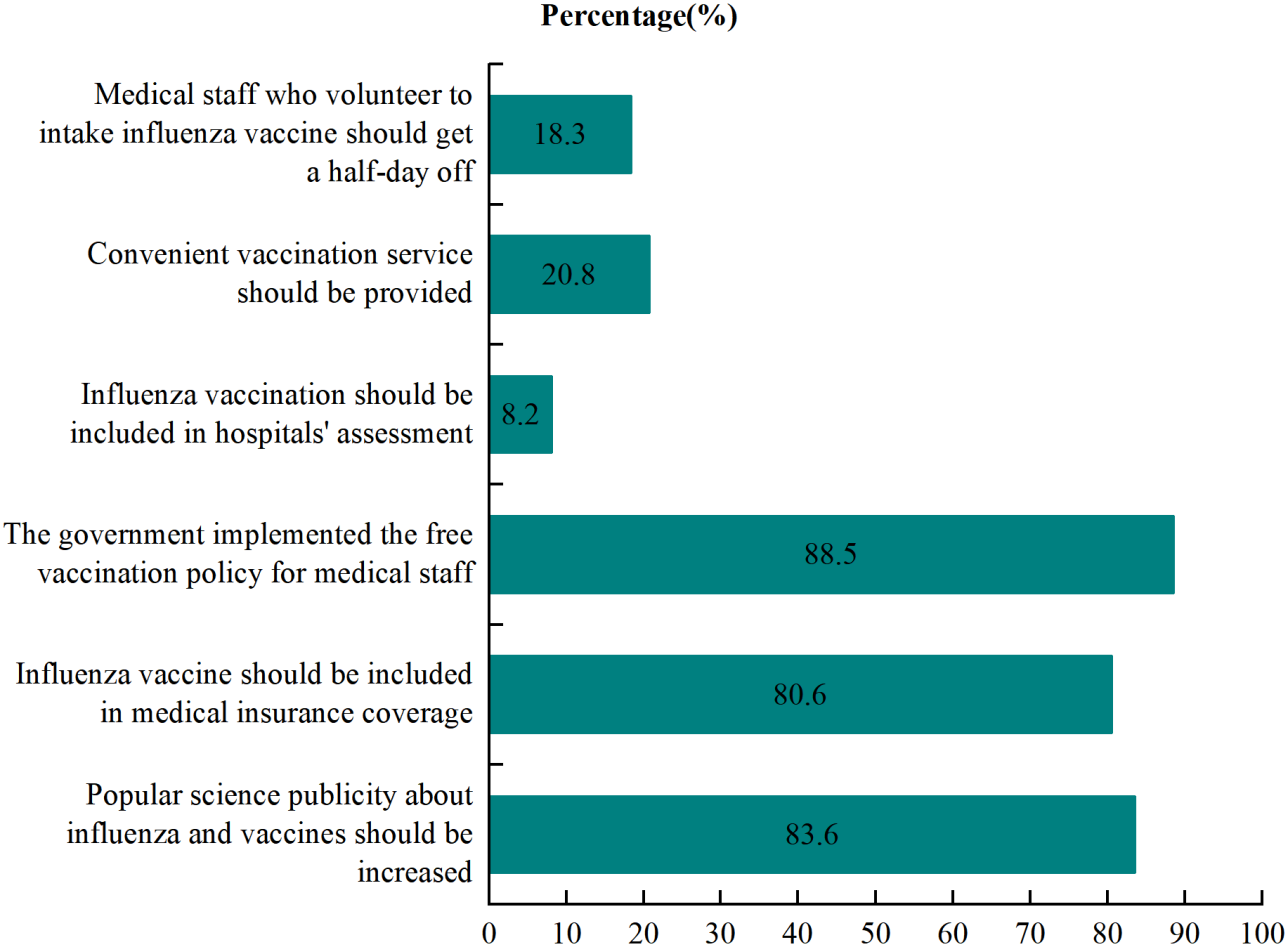
Measures to increase the influenza vaccination rate among community HCWs.

In the bivariate influenza analysis, participants with knowledge scores ≥ 4 (OR: 2.66, 95% CI: 1.42–4.99), without IVH (OR: 4.43, 95% CI: 2.10–9.34), HCWs who were in an active vaccination promoted CHS (OR: 4.39, 95% CI: 2.63–7.33), HCWs with vaccination willingness in the next season (OR: 7.48, 95% CI: 4.11–13.62), and those vaccinated in the 2020/2021 season (OR: 4.17, 95% CI: 2.25–7.70) were more likely to recommend vaccination to their patients. After adjusting for potential confounders in the model, compared with not recommending vaccination to their patients, the adjusted ORs (95% CIs) of the variables are as follows: HCWs with knowledge scores ≥ 4 (adjusted OR: 2.58, 95% CI: 1.30–5.13), without IVH (adjusted OR: 2.33, 95% CI: 1.05–5.14), HCWs who were in an active vaccination promoted CHS (adjusted OR: 2.65, 95% CI: 1.51–4.65), and participants with vaccination willingness in the next influence season (adjusted OR: 4.32, 95% CI: 2.16–8.64), were significantly more likely to recommend influenza vaccination to patients Table 2. The measures proposed by medical staff on how to improve influenza vaccination coverage among community HCWs included: the government should implement a free vaccination policy for medical staff (88.5%), popular science publicity about influenza and vaccines should be increased (83.6%), influenza vaccine should be included in medical insurance coverage (80.6%), and convenient vaccination service should be provided (20.8%) (Fig 3).

## Discussion

To our knowledge, this study is the first investigation in Chongqing, China, that assess influenza vaccination rates among community HCWs during the COVID-19 pandemic. It examined the KAP and perceived barriers and benefits of influenza vaccination of the HCWs. Additionally, we also sought to explore and identify factors associated with influenza vaccination during the current COVID-19 pandemic. The influenza vaccination rate for the 2020–2021 period was 46.2% among our participants. Factors associated with positive vaccination behavior included being female, having prior influenza experience, not having an IVH, and residing in a community that promotes vaccination. HCWs with knowledge scores of ≥4, no IVH, those in a vaccination-promoting community, and those willing to be vaccinated were more likely to recommend vaccination to their patients.

Data from the Chongqing Municipal Immunization and Prevention System, showed relatively low coverage in influenza vaccination among Chongqing HCWs in the past season (2019−2020), compared to the influenza vaccination coverage rate in Chinese respiratory HCWs (67%) [13] and HCWs in Saudi Arabia (67.6%) [29]. The low participation might be the result of lacking influenza vaccination policies, such as mandatory vaccination, but existing issues with vaccine procurement, storage, and distribution, in Chongqing. For example, In the United States, influenza vaccination rates among HCWs ranged from the highest (94.8%) when workplace vaccination was required to the lowest (47.6%) when vaccination was not required, not recommended, or offered at the workplace. Besides, our study manifested that the influenza vaccine uptake among Chongqing HCWs has risen this year (2020−2021). One possible explanation for the increase is that the COVID-19 pandemic has affected HCWs’ willingness to vaccinate against influenza during the current season [30]. Another possible explanation is that the 2020–2021 season was surveyed during the COVID-19 pandemic in mainland China when there was no widely available COVID-19 vaccine. Therefore, most people opted for the seasonal flu vaccine.

In our study, we investigated possible factors that could affect influenza vaccine uptake in the current 2020–2021 flu season. Previous studies stated that the HCWs’ vaccination behaviors were related to complacency, physical availability, affordability, geographical accessibility, and the HCWs’ attitudes toward influenza and influenza vaccines [31–33]. For instance, due to the free vaccination policy and the high availability of vaccines, the coverage of HCWs in Xining City, China has increased significantly [34]. This study presented a similar result. The reluctance of Chongqing HCWs to get vaccinated was mainly yielded from doubts about the necessity of vaccination, the cost of the vaccine, and concerns about the inoculation effect. Therefore, appropriate influenza vaccine policies, including free vaccination and vaccination incentives, and promotion of publicity about influenza and vaccine would be essential to enhance vaccine uptake [35–37].

Consistent with our findings, a recent literature review found that females were more likely to receive vaccine [34,38]. However, another study [39] showed that male respondents had higher vaccine acceptance rates. Interestingly, we found that females did not correlate with a higher vaccination willingness in the next flu season, which could be partly related to a higher level of fear of side effects following vaccination among females, particularly in relation to pregnancy. Similar result was reported in a previous study [40]. Therefore, additional studies are required to explore the link between influenza vaccine acceptance and females. In addition, our study also demonstrated that higher levels of education (university and above education) were associated with increased influenza vaccination willingness. However, no association was found between years of service and vaccine uptake, the recommendation possibility of vaccines, or vaccination willingness, in our study. This result was accordant to the studies that claimed the length of service did not correlate with compliance with vaccination [40,41].

Previous studies have demonstrated that the level of knowledge is positively correlated with vaccine acceptance among HCWs [40,42]. A large multicenter study conducted in France also suggested that the low influenza vaccine coverage reported was associated with a lack of knowledge regarding influenza and its vaccine [43]. However, no significant association was found between knowledge scores and vaccination rates during the 2020–2021 influenza season. This finding may indicate that influenza vaccination behavior is influenced more by external environmental factors (e.g., vaccine trust, access to vaccination, and related policies) than by knowledge alone. For instance, Loulergue et al. noted that doubts about vaccine effectiveness and concerns about potential side effects are significant factors influencing HCWs’ vaccination decisions [44]. Other studies have identified the ease of access to vaccines as a key factor affecting vaccine coverage [45]. A survey in the United States revealed that among healthcare facilities that did not mandate vaccination for HCWs, the vaccination rate was 80.4% in those that offered free vaccine compared to 49% in those that did not [46]. In addition to factors related to HCWs themselves, the policies of healthcare institutions also significantly impact vaccination rates. Studies have shown that influenza vaccination rates can reach as high as 97.8% in healthcare facilities that mandate vaccinations for HCWs [46]. Therefore, to improve vaccination rates among HCWs, it is essential to enhance the dissemination of vaccine-related knowledge to alleviate doubts about the vaccine. At the same time, governments and medical institutions should develop and implement incentive policies, such as providing free vaccinations, to further encourage HCWs to actively participate in vaccination.

Vaccine hesitancy (VH) has been reported as the fuel of the re-emergence of vaccine-preventable diseases and the suppressor of vaccination rates [47]. Consequently, influenza vaccine hesitancy (IVH) would reduce the influenza vaccination coverage [21], which also reflected in this study that IVH would negatively impact HCW uptake, vaccination willingness, and recommendation possibility. Research in Chongqing has demonstrated that IVH would be caused by concerns about safety, efficacy, and side effects of the vaccine, complacency, and vaccination inconvenience [28], which would also affect HCWs’ vaccination behaviors [31–33]. Thus, to diminish IVH and further promote vaccination among HCWs in Chongqing, it would be vital to increase the perception of influenza and its vaccine and provide a more accessible vaccination environment.

An interesting result of this study is that an actively vaccination-promoted community health service center for HCWs had a significantly positive association with the HCW’s vaccination willingness and the possibility of recommendation to patients. This correlation has not been investigated previously, to the best of our knowledge. A possible explanation could be that an actively vaccination-promoted community for HCWs would induce a positive attitude from HCWs towards vaccination, which would further link to improved vaccine acceptance. Consistently, we found that Chongqing HCWs who were willing to receive the vaccine were also more likely to recommend it to their patients. As a result, in the long term, building a vaccination-promoted community health service center would be a key component of pandemic preparedness, both to protect them and to promote vaccination among the public population during a pandemic [26,48].

Several limitations were present in this study. Firstly, the data collection was based on self-reports of practices, beliefs, and knowledge, so these answers might be incorrect due to recall bias, responses could be influenced by social desirability bias. Secondly, the cross-sectional design nature of this study limited our exploration of the causal relationship between influenza vaccination coverage and determinants. Thirdly, although multiple established and potential risk factors for influenza vaccination were adjusted for in the statistical model, unknown confounders by other unmeasured or unknown biological and social factors are still possible, which prevented us from judging the association. In addition, the design of the questionnaire was based on previously relevant literature, and the validity of the questionnaire was sufficient. However we did a stability analysis for the third part of the questionnaire, and the results confirmed the validity and reliability of the questionnaire. Fourthly, attitude toward influenza vaccine of HCWs in our study just including only three questions, this might be insufficient to capture a full spectrum of attitudes. We will expand the scope of questions regarding attitudes in our subsequent research. Finally, the survey could only represent the opinions of a certain percentage of community HCWs from a province in southwest China. Thus, the generality might be hindered, and, hence, the conclusions might not be able to apply to other parts of China. In the future, a nationally representative study should be carried out, based on the proposed survey sample of community HCWs from the 34 governorates of China.

## Conclusions and the way forward

Influenza vaccination coverage among HCWs was unstable. It was 46.2% during the COVID-19 pandemic, which is significantly higher than the rates observed in the past influenza season (2019–2020: 12.9%).

IVH, the cost of the vaccine, and the lack of knowledge and awareness of influenza and its vaccine among HCWs would be the barrier to the vaccination willingness and behaviors and/or the possibility of recommendation to patients, while an actively vaccination-promoted community for HCW would be the enabler. Moreover, lack of knowledge, including doubts about the necessity of vaccination and concerns about safety, efficacy, and side effects of the vaccine, and the cost of the vaccine would also exacerbate IVH. Therefore, to increase the influenza vaccination uptake and further protect HCWs and their patients, it is crucial to propagandize influenza and its vaccine among the HCWs, propose free vaccination policies, and build a vaccination-promoted community.

## Supporting information

S1 Fig Influenza vaccination payment method. (TIF)

S1 File Knowledge, attitudes, and practice survey questionnaire: community healthcare workers regarding the seasonal influenza vaccine. (DOCX)

S2 File Data. (XLSX)
