# Supplementary figures and images for: Uptake, knowledge, attitudes, and practices toward seasonal influenza vaccination among community healthcare workers during the COVID-19 pandemic in Chongqing Municipality, China: A cross-sectional study
Source: PLoS One. 2025 Jul 3;20(7):e0327012. doi: 10.1371/journal.pone.0327012 (PMC12225853; doi:10.1371/journal.pone.0327012)

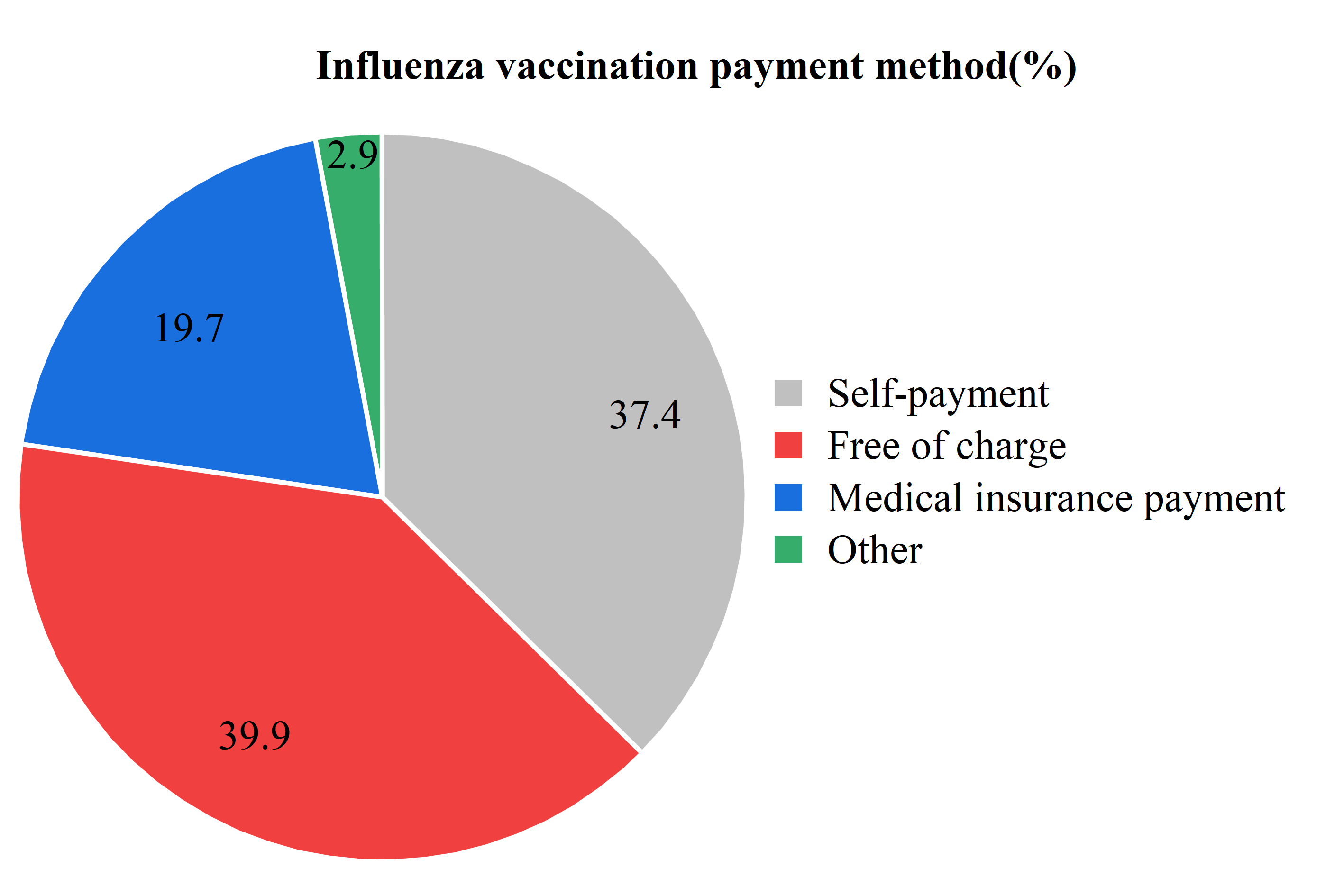

Supplement: S1 Fig — (TIF) [file pone.0327012.s001.tif]
