# Supplementary material for: Uptake, knowledge, attitudes, and practices toward seasonal influenza vaccination among community healthcare workers during the COVID-19 pandemic in Chongqing Municipality, China: A cross-sectional study
Source: PLoS One. 2025 Jul 3;20(7):e0327012. doi: 10.1371/journal.pone.0327012 (PMC12225853; doi:10.1371/journal.pone.0327012)
Supplement: S1 File — (DOCX) [file pone.0327012.s002.docx]

**Knowledge, Attitudes, and Practice Survey Questionnaire: Community healthcare workers regarding the seasonal influenza vaccine**

1. **General and Demographic Questions**
2. **Name of interviewer**: ______________________
3. **Gender:** ☐ male ☐ female
4. **Date of birth:** ....../……/………….
5. **Area of residence:** ☐ urban ☐rural
6. **What is your education level?**
   1. ☐ Middle school or lower
   2. ☐ High/secondary level or lower
   3. ☐ Junior college
   4. ☐ Bachelor
   5. ☐ Postgraduate or higher
7. **Years of medical service:** ____
8. **What is your highest degree major?**
   1. ☐ Clinical medicine
   2. ☐ Traditional Chinese medicine
   3. ☐ Integrative medicine
   4. ☐ Nursing
   5. ☐ Preventive medicine or public health
   6. ☐ Others
9. **What is your professional qualification?**
   1. ☐ Primary title or lower
   2. ☐ Middle title
   3. ☐ Senior title
10. **Knowledge, attitudes, and practice regarding influenza and influenza vaccine**
11. **How about your health condition ?**
    1. ☐ Good
    2. ☐ General
    3. ☐ Fair
    4. ☐ poor
12. **Have you had influenza-like illness (ILI) in the past 3 years ?**
    1. ☐ Yes
    2. ☐ No
    3. ☐ I don’t know
13. **Have you** **had influenza in the past 3 years ?**
    1. ☐ Yes
    2. ☐ No
    3. ☐ I don’t know
14. **Have you asked for leave due to influenza or ILI in the past 3 years?**
    1. ☐ Yes, about days
    2. ☐ No
15. **Did you receive the influenza vaccine in the last flu season?**
    1. ☐ Yes
    2. ☐ No (skip to question 15)
16. **How do you pay for influenza vaccination?**
    1. ☐ Self-paid
    2. ☐ Free
    3. ☐ Medical insurance
    4. ☐ I don’t remember
17. **Do you plan to receive the influenza vaccine in the next flu season?**

a) ☐ Yes

b) ☐ No (skip to question 16)

c） ☐ I don’t know (skip to question 16)

1. **Why don’t you plan to receive the influenza vaccine in the next flu season? (Open -ended question)**
   1. ☐ Concerned about adverse reactions
   2. ☐ Influenza vaccination is unnecessary
   3. ☐ Lack of time during working hours
   4. ☐ Inconvenience vaccination service
   5. ☐ Vaccination fee is not free
   6. ☐ Unknown about contraindications of influenza vaccines
   7. ☐ Concerned about vaccine efficacy
   8. ☐ Doubt about vaccine quality
   9. ☐ Others,
   10. ☐ I don’t remember
2. **Have you recommended your patients receive the influenza vaccine?**
   1. ☐ Yes (skip to question 18)
   2. ☐ No
3. **Why don’t you recommend your patients receive the influenza vaccine? (Multiple choice)**
   1. ☐ Unknown about contraindications of influenza vaccines, and dare not recommend it to patients
   2. ☐ Concerned about patients’ misunderstanding of selling vaccines
   3. ☐ Beyond the scope of HCW’s responsibility
   4. ☐ Uncertainty of vaccine effectiveness to patients
   5. ☐ Patients do not trust the HCW’s recommendations on vaccines
4. **What do you think is the coverage rate of influenza vaccine for the whole population in China?**
   1. ☐ Less than 10%
   2. ☐ 10%~20%
   3. ☐ 20%~30%
   4. ☐ Above 30%
5. **Which of the following 5 statements is correct ?** **(Multiple choice)**
   1. ☐ There is no difference between influenza and cold
   2. ☐ The whole population is susceptible to influenza
   3. ☐ Immunization is an important strategy for preventing influenza（Influenza vaccine can only prevent influenza annually）
   4. ☐ Influenza virus is transmitted mainly by droplets and aerosols originating from the respiratory secretions of infected people and occasionally via contact with virus-contaminated fomites.
   5. ☐ Influenza vaccination can protect themselves as well as their families and patients for HCWs.
6. **As you know, what are the factors that affect the public to receive the flu vaccine ? (Open-ended question)**
   1. ☐ Unknown about influenza vaccines
   2. ☐ Inconvenience vaccination service
   3. ☐ Influenza vaccines price is too high to be acceptable
   4. ☐ It’s unnecessary to receive the influenza vaccine
7. ☐ Others,
8. **Community HCWs should receive influenza vaccine.**
   1. ☐ Yes
   2. ☐ No
9. **Flu vaccines should be covered by public health insurance.**
   1. ☐ Yes
   2. ☐ No
10. **The government should introduce a policy to provide free flu vaccinations for healthcare workers and other eligible groups.**
    1. ☐ Yes
    2. ☐ No
11. **Influenza vaccine hesitancy**
12. **It’s necessary to get an influenza vaccination against influenza ?**
    1. ☐ Strongly agree
    2. ☐ Agree to some extent
    3. ☐ Neutral
    4. ☐ Disagree to some extent

i) ☐ Strongly disagree

1. **The following 9 statements are about seasonal flu and flu vaccine, please state your point of view regarding them.**

|  | **Strongly Agree** | **Agree** | **Neutral** | **Disagree** | **Strongly Disagree** |
| --- | --- | --- | --- | --- | --- |
| a. Flu vaccine is effective |  |  |  |  |  |
| b. Flu vaccine is safe. |  |  |  |  |  |
| c. I worry about the flu vaccine for vaccine incidents. |  |  |  |  |  |
| d. I have a high risk of getting flu. |  |  |  |  |  |
| e. Flu is a big threat to my health. |  |  |  |  |  |
| f. Flu vaccine is necessary to prevent me from getting flu. |  |  |  |  |  |
| g. The traffic from my house to the vaccination clinic is convenient. |  |  |  |  |  |
| h. I can afford the flu vaccine. |  |  |  |  |  |
| i. I can easily find time to the clinic for flu vaccination. |  |  |  |  |  |

**Promotion Measures**

1. **During the influenza season, did your community hospital actively promote influenza vaccination among healthcare workers in the past? [Single-choice question] ***

a) ☐ Yes, promoted, the promotion measures were (fill in the blank)

b）☐ No, not promoted

c） Not clear
